# A mapping review of studies exploring the barriers and facilitators to a dementia diagnosis through an intersectionality lens

**DOI:** 10.1192/bjo.2025.17

**Published:** 2025-04-11

**Authors:** Ben Hicks, Katherine Wheatley, Emma Porter, Nicolas Farina, Sube Banerjee

**Affiliations:** Brighton and Sussex Medical School, University of Sussex, Brighton, UK; Faculty of Health, University of Plymouth, Plymouth, UK; Faculty of Medicine and Health Sciences, University of Nottingham, Nottingham, UK

**Keywords:** Dementias/neurodegenerative diseases, diagnosis, mapping review, intersectionality, social location

## Abstract

**Background:**

Promoting a ‘timely’ diagnosis is a global policy directive.

**Aims:**

This review adopts an intersectional approach, visually mapping the existing literature to highlight gaps in the evidence base on barriers and facilitators to dementia diagnosis.

**Method:**

A systematic approach was undertaken, following the PRISMA guidelines, updating previous reviews. The literature search was conducted on PubMed, PsycINFO, CINAHL Complete and Scopus. In line with mapping review methodology, we report the current state of the literature by describing the number of studies that outline barriers and facilitators to seeking help for a dementia diagnosis, split by social categorisation.

**Results:**

On the 7 June 2024, a total of 45 studies were identified. Our mapping demonstrated the majority of studies were derived from high-income countries and did not specify whether they were exploring barriers and facilitators through a specific social lens. Ethnicity was one of the few social categories where a range of evidence was reported. Other categories, such as socioeconomic status, gender and sexual orientation, received limited research attention.

**Conclusions:**

Our mapping review suggests the large body of work within this field tends to treat people with dementia and their carers as homogenous and androgenous groups. To better inform this key policy directive, studies are needed that explore the influence of social determinants on people’s experiences of seeking a dementia diagnosis. Such work would create a richer, more nuanced evidence base that better elicits ways of addressing inequalities and inequities that arise at this key stage of people’s dementia care journey.

Global policy directives advocate an early or ‘timely’ diagnosis of dementia.^1–3^ Although there are few longitudinal data examining the benefits of these directives,^4,5^ a range of arguments have been put forward. It has been suggested an early/timely diagnosis can help to sustain the well-being of the individual and their family, support people to better manage the condition^6^ and help ensure individuals can uphold their human rights and citizenship.^2,7,8^ Modelling highlights the possibility that an early diagnosis might reduce societal health and social care costs by preventing unnecessary admissions to general hospitals (medium term) and care homes (longer term).^2,9^

Despite efforts to facilitate a timely diagnosis of dementia, research continues to demonstrate the challenges posed in realising this ambition. For instance, underdetection of dementia is common in high- and middle-income countries (60 and 90%, respectively).^10^ Even when detected, nearly half of all people feel diagnosis should have been made earlier.^11^ Research has therefore continued to gather the perspectives of key stakeholders with the aim of elucidating the barriers and facilitators to disclosing, receiving and managing a diagnosis.^7,12–17^ One such review synthesised the past 30 years of research (1986–2017), drawing on 32 studies across 13 countries. It highlighted persistent barriers and facilitators to help-seeking for a dementia diagnosis from the perspectives of people with dementia and carers.^14^ A summary of the review findings is shown in Table 1.

**Table 1:** Summary of identified barriers and facilitators to a dementia diagnosis from Parker et al

Barriers	
Denial	People unwilling to face the possibility that signs and symptoms may be dementia. Could result in carers compensating for difficulties faced by the person rather than seeking help.
Stigma and fear	Real or perceived societal negative attitudes toward dementia or a fear of a dementia diagnosis delayed help-seeking.
Lack of knowledge	Lack of knowledge around the early signs and symptoms of dementia.
Normalisation of symptoms	People attributed early symptoms of dementia to other factors such as ageing, stress and physical and/or psychological health problems. This often occurred in the first year when symptoms were mild.
Preserving autonomy	Concern that a diagnosis would undermine the independence of the person. Carers also respected the wishes of the person not to seek help despite realising there was an issue.
Lack of perceived need	People did not consider the difficulties they faced to be of sufficient problem to seek a diagnosis.
Unaware of changes	People were unaware of any changes that may require help. This was most common in people who lived alone or did not see their carers regularly.
Lack of informal network support	Help-seeking delayed when informal social networks such as friends and family did not support the carer’s desire to seek help.
Carer difficulties	Carers delayed help-seeking because of other life challenges or the perceived impact that a dementia diagnosis would have on their own lives.
Problems accessing help	People were unsure of where to seek help or faced physical (e.g. lack of transport to health services) and/or practical (e.g. cost of health insurance, language challenges) barriers to a diagnosis.

Facilitators	
Recognising symptoms as a problem	Once recognised symptoms as a challenge and could no longer attribute them to another condition then people were more likely to seek help.
Prior knowledge and contacts	Having prior knowledge of dementia or contacts who had knowledge of dementia or worked in health services. Knowledge from the media could also support help-seeking.
Support from informal network	Informal networks providing legitimacy, support and encouragement to the person or their carer to seek help.

## Intersectionality and diagnosis

This work provides a useful overview and understanding of the general barriers and facilitators people may encounter when seeking a dementia diagnosis. Although the authors did not set out to examine the influence of social determinants on this process, they did discuss the role of ‘ethnicity’. However, other sociodemographic determinants were not reported. Established work on ‘intersectionality’ suggests the value of using a broader lens, to understand people holistically as beings who encounter both oppression and privilege as a consequence of their social location.^18^ Research has highlighted how aspects of a person’s social location such as living context,^19,20^ age,^21,22^ gender^23,24^ and sexual orientation,^25,26^ and the intersecting nature of these sociodemographic characteristics,^27,28^ can both positively and adversely influence their wider experiences of living with dementia. In this study, complementary to that conducted by Parker et al,^14^ we aim to map the broad range of social determinants in the literature on help-seeking for a dementia diagnosis, drawing on the concept of intersectionality as a methodological tool. Our primary objective was to develop a visual representation of the data^29^ to enable the identification of gaps in the evidence base and so support future research in this important area.

## Method

### Protocol and registration

The protocol for this mapping review was preregistered on Zenodo, reference number 5179785.^30^ A similar systematic methodology to Parker and colleagues^14^ was adopted. An updated review (2018 to present) sought to elicit additional papers.

### Literature search: eligible studies and inclusion/exclusion criteria

All studies identified in the systematic review by Parker et al,^14^ were included. An updated literature search was conducted on PubMed, PsycINFO, CINAHL Complete and Scopus. Only primary data articles were included, although existing reviews and bibliographies were used to identify any missed literature. An initial search was conducted on the 31 August 2021, and this was updated on the 7 June 2024.

We adapted the search terms identified by Parker et al,^14^ and piloted the search strategies to ensure that all articles included within this original review were also elicited through our own processes. Following this, we limited our search to studies published after the 1 January 2018, when the original review was concluded. Search terms were split into four categories: (a) condition, (b) barriers/facilitators, (c) diagnosis and (d) exclusion terms (see Supplementary Material available at https://doi.org/10.1192/bjo.2025.17).

Criteria for inclusion were as follows: (a) studies with a research aim/question (or questions asked during data collection) that related to barriers or facilitators to help-seeking for a dementia diagnosis, and (b) findings from the perspective of carers (formal or informal) or people subsequently diagnosed with any subtype of dementia. Exclusion criteria for studies were those reporting (a) only demographic characteristics in relation to help-seeking, (b) findings related to help-seeking for care or support post-diagnosis and (b) barriers or facilitators after first contact with a health professional. Studies were not excluded on the basis of study design or outcome measure. Studies did not need to explicitly adopt an ‘intersectional lens’.

### Study selection

Search results were downloaded onto Mendeley software version 2 for Windows (Elsevier, Amsterdam, The Netherlands; https://www.mendeley.com/reference-management/reference-manager/), and duplicates were deleted by N.F. The de-duplicated list was then uploaded onto Rayyan software for Windows (QCRI, Cambridge, MA, USA; https://www.rayyan.ai/),^31^ which allowed for titles and abstracts to be screened by two researchers independently.

Two reviewers (K.W. and E.P.) independently screened 10% of the title and abstracts of the study records to confirm eligibility; reaching a kappa agreement coefficient above our *a priori* threshold of *k* > 0.8 (*k* = 0.84). Any disagreements were discussed and resolved by the whole research team. A single reviewer then independently screened all titles and abstracts for all of the records (K.W.). The full texts of all potentially eligible studies were reviewed by two researchers (B.H. and K.W.) independently, and any disagreements were discussed with a third researcher (N.F.).

### Data abstraction

Data, defined as any information about (or deriving from) a study article were extracted from the full texts by two reviewers (B.H. and K.W.), and checked by the review team. All data were extracted and entered into a pre-piloted form to enable them to be synthesised as part of the mapping review. We did not extract data that were outside the scope of this mapping review (e.g. perceptions or experiences of healthcare professionals). Data that were unclear or not reported were flagged as such, and no efforts were made to seek out this data from original authors. Data items extracted from the eligible studies included: study design, publication date, geographic location, barriers and facilitators for dementia diagnosis, sample size, sample characteristics of participants, and the explicit categorisation from authors about how their findings relate to a social categorisation (e.g. age, gender, ethnicity, sexuality) and whether intersectionality was examined. In accordance with mapping review methodology, we did not appraise the quality or risk of bias of the included studies, as this was not the focus of our mapping review (see Parker et al^14^ for a quality appraisal of the articles included within their review).

### Data synthesis

In accordance with evidence mapping guidelines, the focus of this review was to visualise the findings. A series of figures were developed to highlight the number of studies that reported barriers and facilitators for seeking help for a dementia diagnosis, split by social categorisation. The social categorisations were derived using a bottom-up approach (i.e. based on social categories reported within the study). Two researchers independently coded and grouped categories together (B.H. and N.F.). The exact terminologies for the categories were discussed and decided upon among the research team.

For synthesis, we described studies (i.e. common methodology), articles (i.e. individual publications) and records (i.e. unique explorations of social categories). More specifically, records were conceptualised as (a) an article that uses a unique sample; (b) in instances where multiple articles used the same sample or subsample (i.e. part of a single study reporting findings over multiple articles), these were classified as a single record; and (c) in instances where multiple social categories were explored in a single article, these were counted as multiple records (i.e. double counting).

Microsoft Excel 2024 for Windows and R version 4.4.2 for Windows (R Foundation, Vienna, Austria; https://www.r-project.org/) were used to generate visualisations. This included packages such as rworldmap^32^ and ggplot2 package.^33^

## Results

**Fig. 1:**
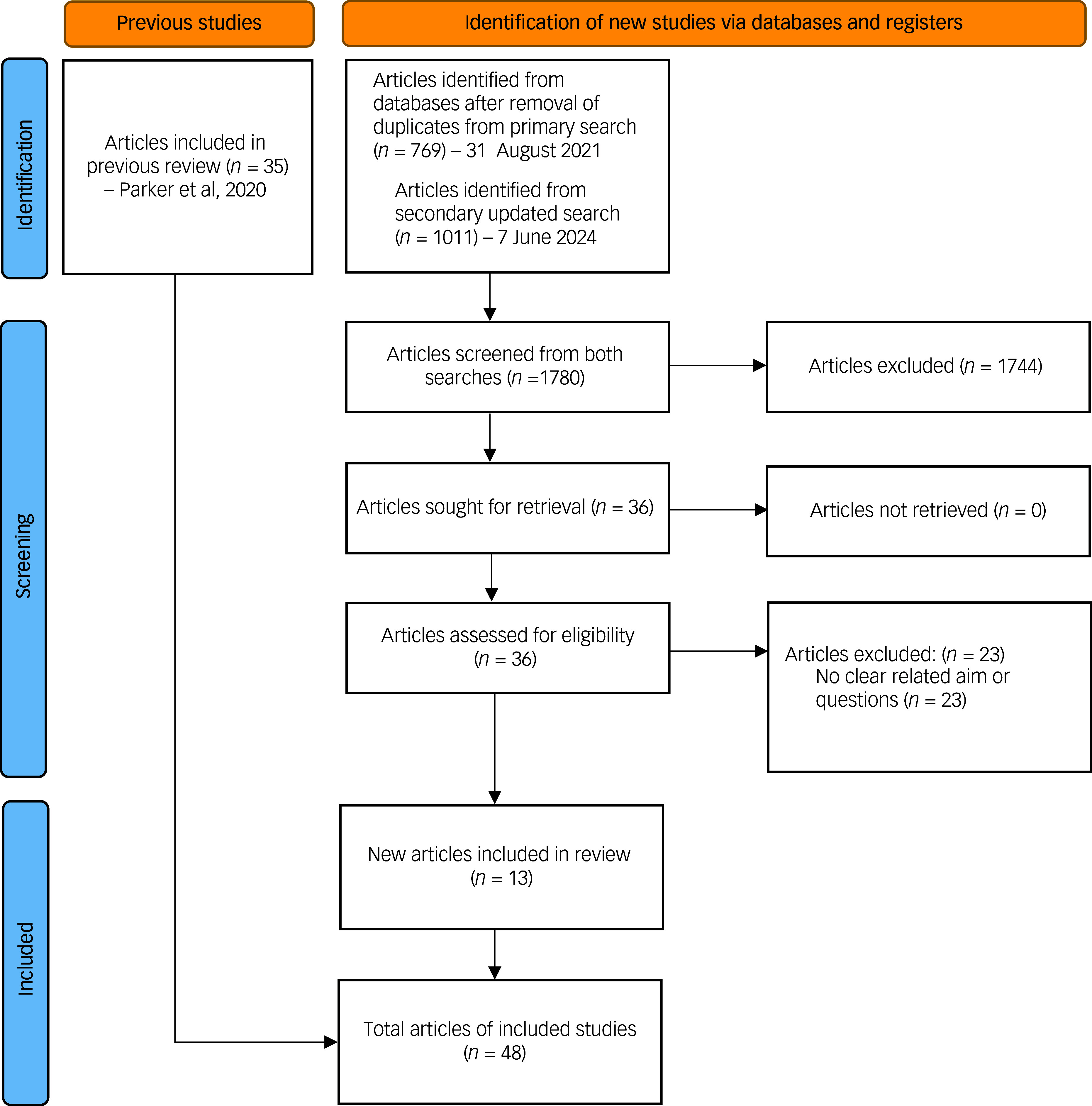
Flow diagram. For more information, visit: http://www.prisma-statement.org/.

Searches conducted on the 31 August 2021 revealed 41 articles (38 studies), and the secondary searches conducted on the 7 June 2024 captured an additional seven articles. A total of 48 articles (45 studies) were included within this mapping review, 13 more than identified in the previous systematic review.^14^ See Fig. 1 for the flow diagram and Supplementary Material for the included studies.

The mean sample size of the included articles was 144 (median 21; minimum 4, maximum 1480), and further details about study characteristics can be found in Table 1. In total, we classified 56 records, with a mean sample size of 143 participants (median 21).

**Fig. 2:**
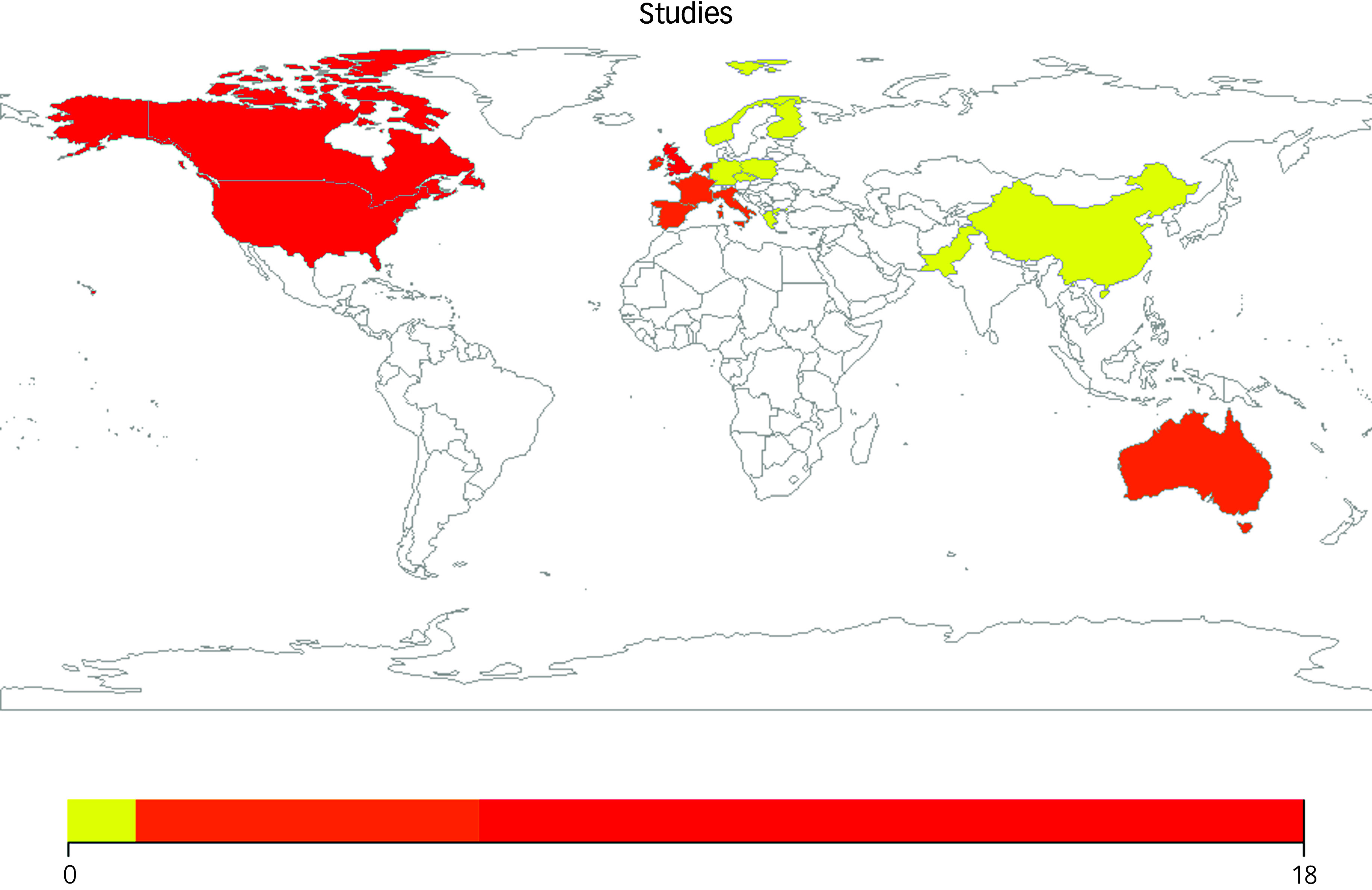
Number of studies identified that related to perceived barriers and facilitators of a dementia diagnosis, split by the country where the data were collected. Studies that recruited across several European countries (Rimmer et al,^43^ Jones et al,^50^ Woods et al,^11^) have been counted multiple times. Yellow, one study; orange, two to six studies; red, seven or more studies.

The research was derived from 18 countries, and all collected data within higher-income settings, with the exception of two, which collected data within China^34^ and Pakistan.^35^ There were no studies from lower-income countries (see Fig. 2).

### Social categorisation

**Fig. 3:**
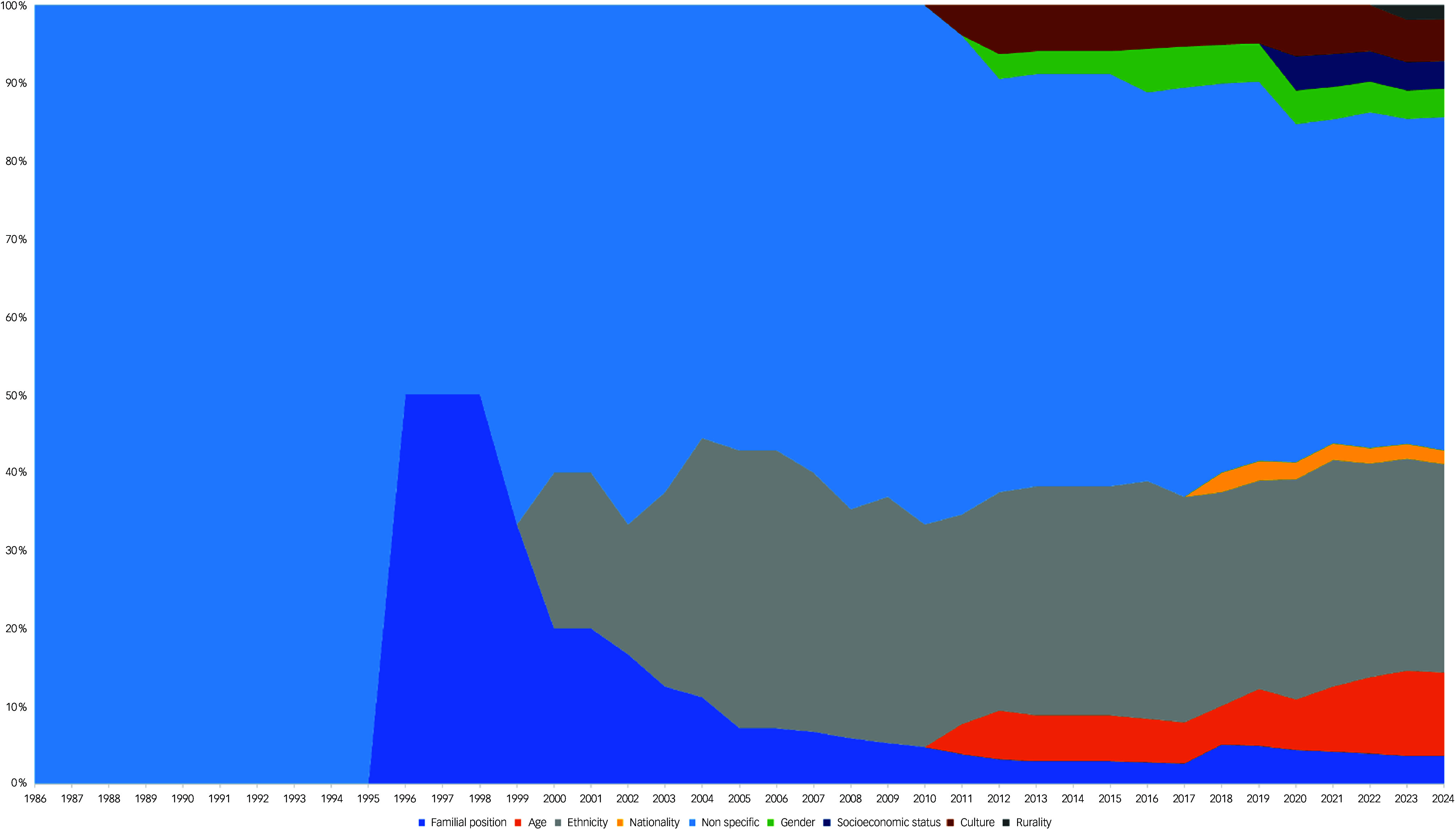
The cumulative proportion of social categories explored per year. Total records (*k* = 56).

Across the included records, the majority did not specify that they were exploring barriers and facilitators through a specific lens (*k* = 24; 42.9%).^34,36–59^ There were, however, several instances whereby recruitment strategies identified a heterogeneous group of participants (e.g. based on rurality), although the authors did not explicitly explore barriers or facilitators through this lens (e.g. Teel and Carson^41^). Ethnicity was the most commonly reported social categorisation (*k* = 15; 26.8%),^35,60–73^ and has prominently featured within the literature since the early 2000s (see Fig. 3). Other identified social categories included age,^21,74–77^ familial position,^11,78^ gender,^67,70^ nationality,^11^ culture,^66,67,71^ rurality^77^ and socioeconomic status.^35^ Of note, we recognise that culture, nationality and ethnicity can be poorly defined and conceptually overlap with one another. In this paper, we have adopted the terminologies used by the original authors, rather than taking a position of the most appropriate term.

Only seven studies explored multiple social categories by design,^11,35,66,67,70,71,77^ with all but two exploring ethnicity alongside one or more other social determinants, including culture,^66,67,71^ age,^67,71^ gender,^67,70^ education^71^ and socioeconomic status.^35^ The remaining studies set out to examine nationality alongside familial position^11^ and age alongside rurality; although the latter study did not appear to achieve these aims.^77^

### Barriers and facilitators

The most frequently reported barriers were the normalisation of symptoms (*k* = 48; 85.7%),^11,21,34,37–40,42–47,49–57,59,61,63–78^ denial/resistance from the person with dementia (*k* = 28; 50.0%)^11,21,37–39,43,44,47,48,50–52,57,63–66,69,70,74–79^ and lack of need (*k* = 26; 46.4%).^34,38–40,43–45,47–50,52,53,55,57,63,66,68–71,75,78^ Carer difficulties were the least frequently reported barrier (*k* = 7, 12.5%).^37,39,40,46,53,57,70^ Only three facilitators were described, and included recognition of accumulating symptoms (*k* = 45, 80.4%),^21,34–46,48–58,61,63–70,72–77^ support from informal networks (*k* = 24, 42.9%)^35,46,49,51–53,61,65,67–72,74–76^ and prior knowledge and contacts (*k* = 21, 37.5%).^34,35,39,40,42,46,52,55,57,61,62,64,67,71,72,74^ No new barriers or facilitators were identified in this review other than those previously outlined by Parker et al.^14^ See Supplementary Table 2 for a breakdown of barriers and facilitators by social lens.

### Intersectionality: perceived barriers

Across the included records, the majority explored perceived barriers within a homogenous group of participants (i.e. non-specific lens) (*k* = 24; 42.9%). It is important to note that only two studies adopted an intersectional approach when examining barriers;^67,71^ however, our mapping identified a number of social categories. The most frequently reported social categorisation for perceived barriers was ethnicity (*k* = 14, 25.0%).^60–73^ With the exception of being unaware of changes and carer difficulties, which were derived from a single study,^70^ all other barriers had been explored across multiple records (*k* ≥ 4) through the lens of ethnicity.

**Fig. 4:**
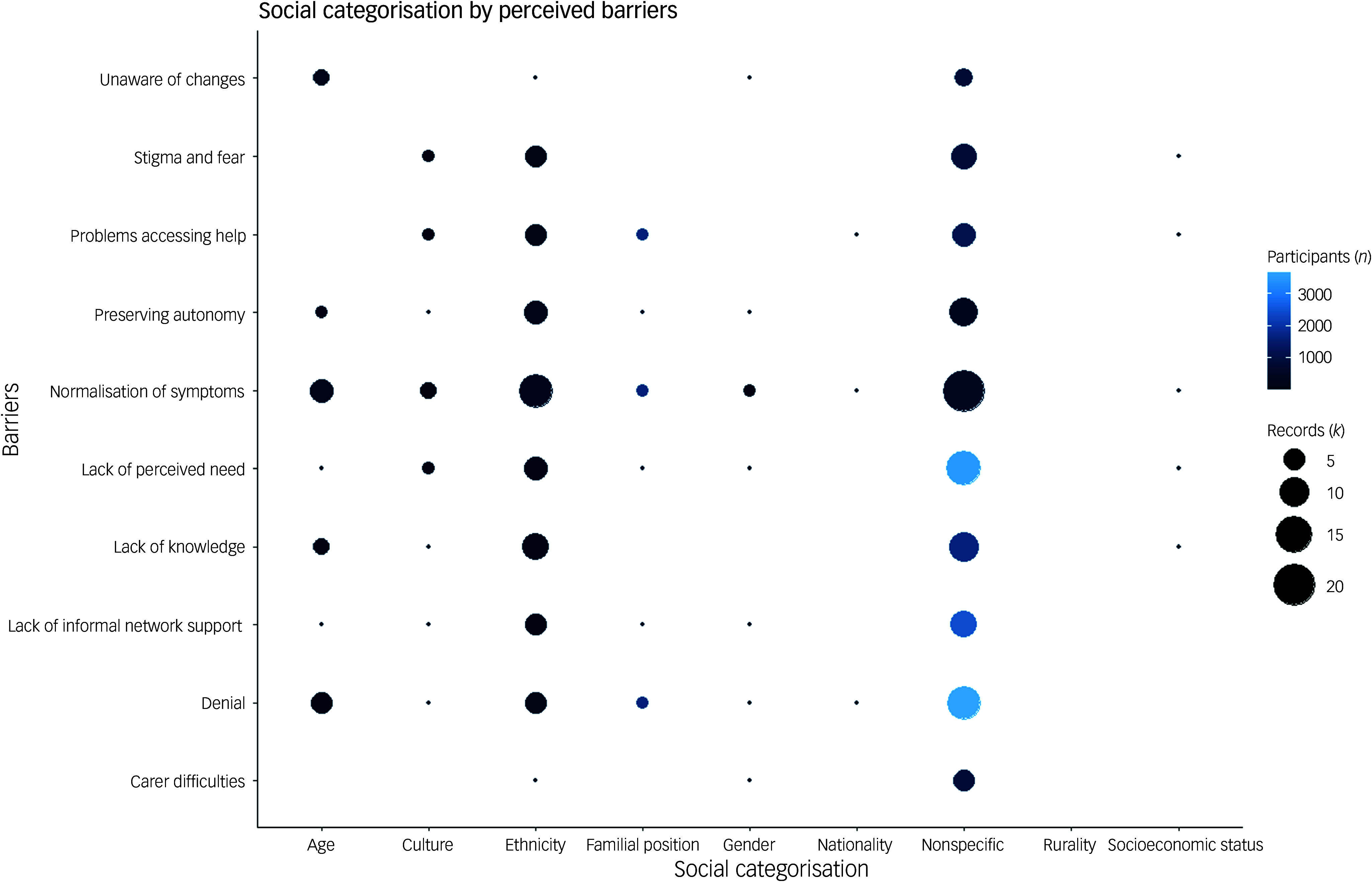
Records identified in which different social categorisations are plotted against perceived barriers to a dementia diagnosis.

Records that focused on the social categories of culture,^66,67,71^ nationality,^11^ socioeconomic status,^35^ familial position^11,78^ and gender^67,70^ tended to identify a range of barriers; however, these were typically derived from a small number of records for each (*k* ≤ 5). Only one study set out to explore the impact of barriers through the lens of rurality,^77^ although the findings did not appear to align with this aim. Irrespective of social categorisation, outside of a few notable exceptions,^11,78^ perceived barriers tended to be explored in smaller sampled qualitative research. See Fig. 4 for a summary.

### Intersectionality: perceived facilitators

**Fig. 5:**
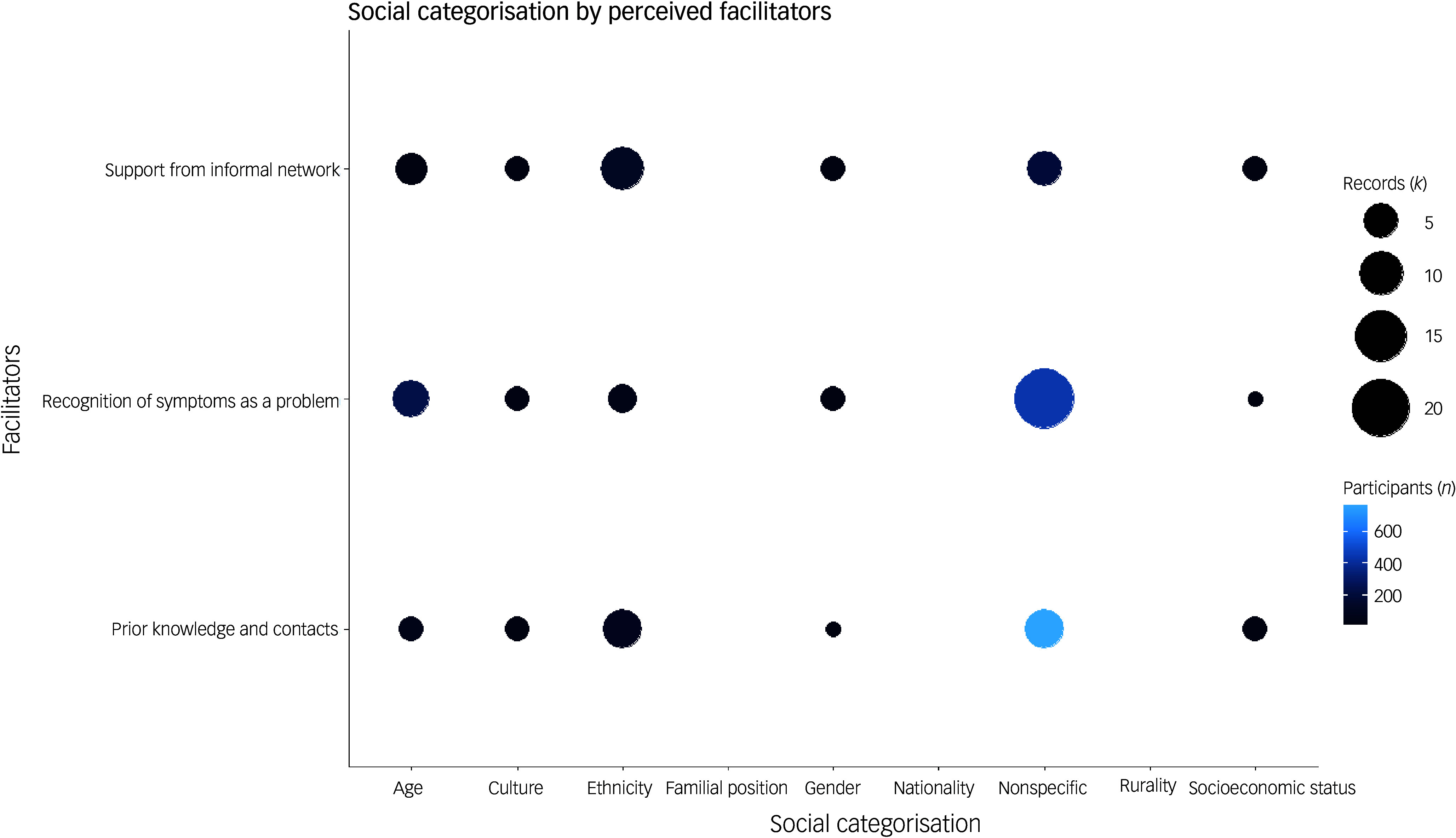
Records identified in which different social categorisations are plotted against perceived facilitators to a dementia diagnosis.

Facilitators were most frequently explored using a non-specific social categorisation (*k* = 23; 41.1%). Again, similar to perceived barriers, our mapping only found the same two studies that adopted an intersectionality lens to examine the facilitators to help-seeking,^67,71^ although a number of social categories were evident in the literature. Across other identified social categorisations, there was a tendency to have a breadth of facilitators explored. There was at least one record that explored each facilitator through the lens of age,^21,67,74–77^ gender,^67,70^ culture,^66,67,71^ ethnicity^35,61–73^ and socioeconomic status.^35^ As with the perceived barriers, ethnicity was once again the most adopted social lens for perceived facilitators (*k* = 14; 25.0%).^35,61–73^ There was also a tendency for studies to explore these social categories within smaller qualitative studies. The largest of these studies (*n* = 92) was a qualitative study that explored facilitators through the social lens of age (early-onset dementia).^75^ Out of the social categorisations identified, only nationality, rurality and familial position had not been explored in terms of the perceived facilitators to a dementia diagnosis. See Fig. 5 for a summary.

## Discussion

Informed by the concept of intersectionality, our review has updated and expanded on the work of Parker et al^14^ by mapping and visually representing the varying social categorisations accounted for in studies that explored people with dementia and their carers’ experiences of seeking a dementia diagnosis. Our findings suggest that the large majority of research within the field regards people with dementia and their carers as a homogenous and androgynous group. It is positive that more recent studies have sought to examine the influence of certain social determinants, particularly ethnicity, culture and age. Even when studies recruit a heterogeneous sample (or subgroup), this appeared a consequence of recruitment strategies, rather than a purposeful attempt to explore the role of different social categories within the study. There is a clear need to develop our understanding of barriers and facilitators of dementia diagnosis through different social lenses.

Although some of the studies in our mapping review looked at multiple social categories, only two^67,71^ drew on an intersectionality lens to elicit an understanding of the interplay between different sociodemographic determinants and their influence on help-seeking for a dementia diagnosis. Czapka and Sagbakken^71^ adopted an intersectionality lens when interpreting their findings, to discuss the interplay between ethnicity, culture and education on the varying experiences of ethnic minorities within the Norwegian dementia care system. Only Koehn et al^67^ employed this lens throughout their study design, analysis and discussion. Using an intersectionality lens to guide research design from the outset could encourage researchers to consider the social positions of privilege and oppression that participants may occupy, and so ensure that these voices are adequately represented through their sampling strategy and analysis procedures. This approach should enable deeper insights into potential barriers and facilitators for help-seeking behaviour, and provide more informed recommendations for policy and practice.

Our mapping review found ethnicity was the most frequently adopted social categorisation. It is clear that ethnicity has a profound impact on accessing dementia health services and determining outcomes.^80–83^ However, future research should be cautious about treating ethnicity as a single dimension, as it may overlook unique historical and social cultural dynamics. Exploring facilitators and barriers to diagnosis through the lens of majority ethnic groups in Pakistan, for example,^35^ is profoundly different from the facilitators and barriers experienced by minority ethnic groups^66^ within a country like the UK. In part, this is attributable to whether the health and support systems are adequately tailored to minority groups. It was not always clear that there was separation of nationality, culture and ethnicity.

Our mapping highlighted significant gaps within the present literature and social categories that were underrepresented. Most notably, current understanding of facilitators and barriers to seeking a diagnosis of dementia have been drawn predominantly from studies conducted within high-income countries, with only two exceptions.^34,35^ This gap should be addressed, given that around 71% of people with dementia will reside in low- and middle-income countries by 2050,^84^ resulting in substantial economic cost.^85^ Research examining help-seeking for a dementia diagnosis in low- and middle-income countries would enable culturally specific and tailored policy planning to occur. Our mapping highlighted that the influence of socioeconomic status (or class) has only been examined in one study,^35^ and other common social categories, such as gender, sexual orientation and rurality, have received little or no attention. It is unsurprising that research on gender and sexual orientation is limited, given that the dementia care agenda is only now acknowledging the influence of these social determinants on people’s experiences.^26,86^ However, the influence of the geo-socio-cultural rural environment on people’s experiences of living with dementia and accessing informal and formal support services has long been established.^19,20,87^ The limited number of studies found during our mapping may be a consequence of our inclusion/exclusion criteria, but may also point to the need for more specific research that examines the interplay of rurality and other intersections on people’s experiences of help-seeking for a dementia diagnosis. Our findings highlighting the gaps in current research also have implications for policy and practice within dementia care. To achieve policy aims of supporting people to access a ‘timely diagnosis’,^2^ improving dementia education more broadly and creating inclusive societies for those living with the condition,^88^ it is important that we are able to draw from evidence that understands these more nuanced accounts. This would enable policy and practice to address better the inequalities and inequities that can be encountered in these early stages of a person’s dementia journey.

### Strengths and limitations

We adopted a pragmatic method by using literature identified in a previous systematic review and then running a new search to identify more recent studies. Therefore, this might be best classified as an update.^89^ Such an approach has benefits in being more efficient on researcher time. Notably, the validity of the inclusion of research from the previous review is dependent on consistent application of criteria. Efforts to optimise and increase transparency of the review process were made through publishing the protocol.^30^ The use of intersectionality in the mapping review adds complementary analyses to the original review, and was intended as a novel way to identify gaps in the current evidence base, particularly from perspectives of those that may be marginalised in the diagnostic process. This approach is similar to that applied in public health, whereby social identities are considered ‘multiple and intersecting’. People from historically oppressed and marginalised groups are focal points, and intersectionality can help reveal disparate health outcomes.^90^ Despite the value of such an approach, we should be vigilant about the shortcomings of adopting an intersectionality lens in future research. For example, it might (a) promote tokenistic recruitment strategies, (b) lead to the view that the experiences and perceptions of a few members of a group reflect the larger population as a whole, (c) promote a hierarchy of researched groups and (d) reduce the complexities of humans into a framework. Such shortcomings should not prevent researchers from attempting to adopt an intersectionality approach, but rather critically reflect on its value within the literature. As mentioned previously, it is important to recognise that social categories were derived based on aims and objectives of the primary study. There are instances where studies had recruited participants from a certain social category (e.g. urban), but were classified as being non-specific because it was not a line of enquiry within the research. We also acknowledge that this review does not explore why certain social groups experience different barriers and facilitators; mapping reviews are often likened to a scoping review, where gaps in evidence are identified, albeit with an emphasis on tabulating findings.^91^ Another limitation is that we double-counted records in our synthesis where multiple social categorisations were explored, thus inflating the number of records and sample sizes. Finally, the adoption of a single researcher screening was chosen for pragmatic reasons. Single reviewer screening can lead to missing studies when compared with double screening (median missed 5% of studies),^92^ and therefore it is possible that our approach may have missed a small proportion of the literature. To minimise the potential of human error and bias, we ensured that 10% of titles/abstracts were double screened. In line with the protocol, we only stopped double screening because agreement surpassed our threshold (*k* = 0.8).

In summary, supporting a timely diagnosis of dementia is a global policy priority. Key to successfully realising this agenda is a need to better understand the barriers and facilitators encountered by people when seeking help for a diagnosis. Complementing the work of Parker et al,^14^ this mapping review highlights that there is substantial literature within this field. However, in most instances, data are derived from groups of participants who are positioned as homogenous and androgynous, without considering their varied and intersecting sociodemographic characteristics. Future research is needed that accounts for the varied social locations a person can occupy and their influence on help-seeking for a dementia diagnosis – particularly in regards to socioeconomic status, gender and sexual orientation. These studies would help build a richer and more nuanced evidence base that is better positioned to inform this important component of dementia policy and care.

## Supporting information

Hicks et al. supplementary material Hicks et al. supplementary material

## Data Availability

The data that support the findings of this study are available from the corresponding author, B.H., upon reasonable request.

## Supplementary material

The supplementary material for this article can be found at https://doi.org/10.1192/bjo.2025.17

## References

[1] World Health Organization. Towards a Dementia Plan: A WHO Guide. WHO, 2018 (https://www.who.int/publications/i/item/9789241514132).

[2] Alzheimer’s Disease International. World Alzheimer Report 2011: The Benefits of Early Diagnosis and Intervention. Alzheimer’s Disease International, 2011 (https://www.alzint.org/resource/world-alzheimer-report-2011/).

[3] Alzheimer’s Disease International. World Alzheimer Report 2021: Journey Through the Diagnosis of Dementia. Alzheimer’s Disease International, 2021 (https://www.alzint.org/u/World-Alzheimer-Report-2021.pdf).

[4] Farina N , Hicks B , Baxter K , Birks Y , Brayne C , Dangoor M , et al. DETERMinants of quality of life, care and costs, and consequences of INequalities in people with Dementia and their carers (DETERMIND): a protocol paper. Int J Geriatr Psychiatry 2020; 35: 290–301. 31876069 10.1002/gps.5246

[5] Couch E , Mueller C , Perera G , Lawrence V , Prina M. The association between an early diagnosis of dementia and secondary health service use. Age Ageing 2021; 50: 1277–82. 34057464 10.1093/ageing/afab079 PMC8837821

[6] Couch E. What are the Benefits of Diagnosing Dementia Early? A Mixed Methods Study. King’s College London, 2021 (https://kclpure.kcl.ac.uk/portal/en/studentTheses/what-are-the-benefits-of-diagnosing-dementia-early-a-mixed-method).

[7] Dubois B , Padovani A , Scheltens P , Rossi A , Dell’Agnello G. Timely diagnosis for Alzheimer’s disease: a literature review on benefits and challenges. J Alzheimer’s Dis 2016; 49: 617–31. 26484931 10.3233/JAD-150692 PMC4927869

[8] Brayne C , Kelly S. Against the stream: early diagnosis of dementia, is it so desirable? BJPsych Bull 2019; 43: 123–5. 30606274 10.1192/bjb.2018.107 PMC8058847

[9] Banerjee S , Wittenberg R. Clinical and cost effectiveness of services for early diagnosis and intervention in dementia. Int J Geriatr Psychiatry 2009; 24: 748–54. 19206079 10.1002/gps.2191

[10] Lang L , Clifford A , Wei L , Zhang D , Leung D , Augustine G , et al. Prevalence and determinants of undetected dementia in the community: a systematic literature review and a meta-analysis. BMJ Open 2017; 7: e011146. 10.1136/bmjopen-2016-011146 PMC5293981 28159845

[11] Woods B , Arosio F , Diaz A , Gove D , Holmerová I , Kinnaird L , et al. Timely diagnosis of dementia? Family carers’ experiences in 5 European countries. Int J Geriatr Psychiatry 2019; 34: 114–21. 30246266 10.1002/gps.4997 PMC6586062

[12] Bamford C , Lamont S , Eccles M , Robinson L , May C , Bond J. Disclosing a diagnosis of dementia: a systematic review. Int J Geriatr Psychiatry 2004; 19: 151–69. 14758581 10.1002/gps.1050

[13] Bunn F , Goodman C , Sworn K , Rait G , Brayne C , Robinson L , et al. Psychosocial factors that shape patient and carer experiences of dementia diagnosis and treatment: a systematic review of qualitative studies. PLoS Med 2012; 9: e1001331. 23118618 10.1371/journal.pmed.1001331 PMC3484131

[14] Parker M , Barlow S , Hoe J , Aitken L. Persistent barriers and facilitators to seeking help for a dementia diagnosis. Int Psychogeriatr 2020; 32: 611–34. 10.1017/S1041610219002229 32024558

[15] Poyser CA , Tickle A. Exploring the experience of the disclosure of a dementia diagnosis from a clinician, patient and carer perspective: a systematic review and meta-ethnographic synthesis. Aging Mental Health 2019; 23: 1605–15. 30430858 10.1080/13607863.2018.1506747

[16] Robinson L , Gemski A , Abley C , Bond J , Keady J , Campbell S , et al. The transition to dementia – individual and family experiences of receiving a diagnosis: a review. Int Psychogeriatr 2011; 23: 1026–43. 21281553 10.1017/S1041610210002437

[17] Werner P , Goldstein D , Karpas DS , Chan L , Lai C. Help-seeking for dementia: a systematic review of the literature. Alzheimer Dis Assoc Disord 2014; 28: 299–310. 25321607 10.1097/WAD.0000000000000065

[18] Calasanti TM. Incorporating diversity: meaning, levels of research, and implications for theory. Gerontologist 1996; 36: 147–56. 8920083 10.1093/geront/36.2.147

[19] Hicks B , Innes A , Nyman SR. Experiences of rural life among community-dwelling older men with dementia and their implications for social inclusion. Dementia 2021; 20: 444–63. 31718267 10.1177/1471301219887586

[20] Innes A , Morgan D , Kostineuk J. Dementia care in rural and remote settings: a systematic review of informal/family caregiving. Maturitas 2011; 68: 34–46. 21093996 10.1016/j.maturitas.2010.10.002

[21] Novek S , Menec VH. Age, dementia, and diagnostic candidacy: examining the diagnosis of young onset dementia using the candidacy framework. Qual Health Res 2021; 31: 498–511. 33213257 10.1177/1049732320970199

[22] O’Malley M , Carter J , Stamou V , LaFontaine J , Oyebode J , Parkes J. Receiving a diagnosis of young onset dementia: a scoping review of lived experiences. Aging Mental Health 2021; 25: 1–12. 31647324 10.1080/13607863.2019.1673699

[23] Hicks B , Innes A , Nyman SR. Exploring the ‘active mechanisms’ for engaging rural-dwelling older men with dementia in a community technological initiative. Ageing Soc 2020; 40: 1906–38.

[24] Sandberg LJ. ‘I was the woman, he was the man’: dementia, recognition, recognisability and gendered subjectivity. Human Soc Sci Commun 2021; 8: 1–11.

[25] McParland J , Camic PM. How do lesbian and gay people experience dementia? Dementia 2018; 17: 452–77. 27165984 10.1177/1471301216648471

[26] Smith L , Chesher I , Fredriksen-Goldsen K , Ward R , Phillipson L , Newman CE . et al. Investigating the lived experience of LGBT+ people with dementia and their care partners: a scoping review. Ageing Soc 2024; 44: 843–66.

[27] Hulko W. From ‘not a big deal’ to ‘hellish’: experiences of older people with dementia. J Aging Stud 2009; 23: 131–44.

[28] Hulko W. LGBT* individuals and dementia: an intersectional approach. In Lesbian, Gay, Bisexual and Trans* Individuals Living with Dementia (eds S Westwood, E Price): 35–50. Routledge, 2016.

[29] Miake-Lye IM , Hempel S , Shanman R , Shekelle PG. What is an evidence map? A systematic review of published evidence maps and their definitions, methods, and products. Syst Rev 2016; 5: 1–21. 26864942 10.1186/s13643-016-0204-x PMC4750281

[30] Farina N , Razaghi L , Hicks B. A mapping review of barriers and facilitators to a dementia diagnosis adopting an intersectionality lens: a protocol paper. Zenodo 2021. Available from: https://www.academia.edu/80471990/A_mapping_review_of_barriers_and_facilitators_to_a_dementia_diagnosis_adopting_an_intersectionality_lens_A_protocol_paper. 10.1192/bjo.2025.17 PMC12052574 40214113

[31] Ouzzani M , Hammady H , Fedorowicz Z , Elmagarmid A. Rayyan – a web and mobile app for systematic reviews. Syst Rev 2016; 5: 1–10. 27919275 10.1186/s13643-016-0384-4 PMC5139140

[32] South A. rworldmap: a new R package for mapping global data. R J 2011; 3: 35–43.

[33] Wickham H. ggplot2: Elegant Graphics for Data Analysis. Springer, 2009.

[34] Lian Y , Xiao LD , Zeng F , Wu X , Wang Z , Ren H. The experiences of people with dementia and their caregivers in dementia diagnosis. J Alzheimer’s Dis 2017; 59: 1203–11. 28731450 10.3233/JAD-170370

[35] Willis R , Zaidi A , Balouch S , Farina N. Experiences of people with dementia in Pakistan: help-seeking, understanding, stigma, and religion. Gerontologist 2020; 60: 145–54. 30452635 10.1093/geront/gny143

[36] Chenoweth B , Spencer B. Dementia: the experience of family caregivers. Gerontologist 1986; 26: 267–72. 3721234 10.1093/geront/26.3.267

[37] Boise L , Morgan DL , Kaye J , Camicioli R. Delays in the diagnosis of dementia: perspectives of family caregivers. Am J Alzheimer’s Dis 1999; 14: 20–6.

[38] Knopman D , Donohue JA , Gutterman EM. Patterns of care in the early stages of Alzheimer’s disease: impediments to timely diagnosis. J Am Geriatr Soc 2000; 48: 300–4. 10733057 10.1111/j.1532-5415.2000.tb02650.x

[39] Wackerbarth SB , Johnson MM. The carrot and the stick: benefits and barriers in getting a diagnosis. Alzheimer Dis Assoc Disord 2002; 16: 213–20. 12468895 10.1097/00002093-200210000-00002

[40] Streams ME , Wackerbarth SB , Maxwell A. Diagnosis-seeking at subspecialty memory clinics: trigger events. Int J Geriatr Psychiatry 2003; 18: 915–24. 14533124 10.1002/gps.946

[41] Teel CS , Carson P. Family experiences in the journey through dementia diagnosis and care. J Family Nurs 2003; 9: 38–58.

[42] Krull AC. First signs and normalizations: Caregiver routes to the diagnosis of Alzheimer’s disease. J Aging Stud 2005; 19: 407–17.

[43] Rimmer E , Wojciechowska M , Stave C , Sganga A , O’Connell B. Implications of the Facing Dementia Survey for the general population, patients and caregivers across Europe. Int J Clin Pract 2005; 59: 17–24. 15801187 10.1111/j.1368-504x.2005.00482.x

[44] Bond J , Stave C , Sganga A , Vincenzino O , O’Connell B , Stanley R. Inequalities in dementia care across Europe: key findings of the Facing Dementia Survey. Int J Clin Pract 2005; 59: 8–14. 10.1111/j.1368-504x.2005.00480.x 15801185

[45] Eustace A , Bruce I , Coen R , Cunningham C , Walsh C , Walsh JB , et al. Behavioural disturbance triggers recognition of dementia by family informants. Int J Geriatr Psychiatry J Psychiatry 2007; 22: 574–9. 10.1002/gps.1718 17136712

[46] Carpentier N , Ducharme F , Kergoat M-J , Bergman H. Social representations of barriers to care early in the careers of caregivers of persons with Alzheimer’s disease. Res Aging 2008; 30: 334–57.

[47] Speechly CM , Bridges-Webb C , Passmore E. The pathway to dementia diagnosis. Med J Austr 2008; 189: 487–9. 10.5694/j.1326-5377.2008.tb02140.x 18976188

[48] Tsolaki M , Paraskevi S , Degleris N , Karamavrou S. Attitudes and perceptions regarding Alzheimer’s disease in Greece. Am J Alzheimer’s Dis Other Demen 2009; 24: 21–6. 19047472 10.1177/1533317508325990 PMC10846124

[49] Carpentier N , Bernard P , Grenier A , Guberman N. Using the life course perspective to study the entry into the illness trajectory: the perspective of caregivers of people with Alzheimer’s disease. Soc Sci Med 2010; 70: 1501–8. 20207459 10.1016/j.socscimed.2009.12.038 PMC5123874

[50] Jones RW , Mackell J , Berthet K , Knox S. Assessing attitudes and behaviours surrounding Alzheimer’s disease in Europe: key findings of the Important Perspectives on Alzheimer’s Care and Treatment (IMPACT) survey. J Nutri Health Aging 2010; 14: 525–30. 10.1007/s12603-010-0263-y PMC12880341 20818466

[51] Leung KK , Finlay J , Silvius JL , Koehn S , McCleary L , Cohen CA , et al. Pathways to diagnosis: exploring the experiences of problem recognition and obtaining a dementia diagnosis among Anglo-Canadians. Health Soc Care Commun 2011; 19: 372–81. 10.1111/j.1365-2524.2010.00982.x 21223398

[52] Manthorpe J , Samsi K , Campbell S , Keady J , Watts S , Gemski A , et al. The Transition from Cognitive Impairment to Dementia: Older People’s Experiences . King’s College London, 2011 (https://eprints.kingston.ac.uk/id/eprint/18322/2/SDO_ES_08-1809-229_V01.pdf).

[53] Chrisp TA , Tabberer S , Thomas BD , Goddard WA. Dementia early diagnosis: triggers, supports and constraints affecting the decision to engage with the health care system. Aging Mental Health 2012; 16: 559–65. 22360880 10.1080/13607863.2011.651794

[54] Bunn F , Goodman C , Sworn K , Rait G , Brayne C , Robinson L , et al. Psychosocial factors that shape patient and carer experiences of dementia diagnosis and treatment: a systematic review of qualitative studies. PLoS Med 2012; 9: e1001331. 10.1371/journal.pmed.1001331 PMC3484131 23118618

[55] Feldman L , Wilcock J , Thuné-Boyle I , Iliffe S. Explaining the effects of symptom attribution by carers on help-seeking for individuals living with dementia. Dementia 2017; 16: 375–87. 26130674 10.1177/1471301215593185

[56] Brady AM , Coughlan B , Clarke N , Edgeworth J. ‘If it happens to me, I would want to know earlier’: a qualitative exploration of views on timing of dementia diagnosis within a specific geographical region in Ireland. Clinical Psychology Today, 11 Nov 2022 (https://clinicalpsychologytoday.wordpress.com/2022/11/11/if-it-happens-to-me-i-would-want-to-know-earlier-a-qualitative-exploration-of-views-on-timing-of-dementia-diagnosis-within-a-specific-geographical-region-of-ireland/).

[57] Parker M , Barlow S , Hoe J , Aitken LM. The bubble of normalisation: a qualitative study of carers of people with dementia who do not seek help for a diagnosis. J Geriatr Psychiatry Neurol 2022; 35: 717–32. 34951319 10.1177/08919887211060018 PMC9386763

[58] Sideman AB , Gilissen J , Harrison KL , Garrett SB , Terranova MJ , Ritchie CS , et al. Caregiver experiences navigating the diagnostic journey in a rapidly progressing dementia. J Geriatr Psychiatry Neurol 2023; 36: 282–94. 36412170 10.1177/08919887221135552 PMC10265278

[59] Acton DJ , Jaydeokar S , Taylor R , Jones S. Exploring the lived experiences and care challenges of formal paid caregivers for people with intellectual disability and dementia. J Intell Disabil [Epub ahead of print] 30 May 2024. Available from: 10.1177/17446295241259076. 38816805

[60] Ortiz F , Fitten LJ. Barriers to healthcare access for cognitively impaired older Hispanics. Alzheimer Dis Assoc Disord 2000; 14: 141–50. 10994655 10.1097/00002093-200007000-00005

[61] Cloutterbuck J , Mahoney DF. African American dementia caregivers: the duality of respect. Dementia 2003; 2: 221–43.

[62] Zhan L. Caring for family members with Alzheimer’s disease: perspectives from Chinese American caregivers. J Gerontol Nurs 2004; 30: 19–29. 10.3928/0098-9134-20040801-06 15359526

[63] Clark PC , Kutner NG , Goldstein FC , Peterson‐Hazen S , Garner V , Zhang R , et al. Impediments to timely diagnosis of Alzheimer’s disease in African Americans. J Am Geriatr Soc 2005; 53: 2012–7. 16274388 10.1111/j.1532-5415.2005.53569.x

[64] Neary SR , Mahoney DF. Dementia caregiving: the experiences of Hispanic/Latino caregivers. J Transcult Nurs 2005; 16: 163–70. 15764640 10.1177/1043659604273547

[65] Hughes T , Tyler K , Danner D , Carter A. African American caregivers: an exploration of pathways and barriers to a diagnosis of Alzheimer’s disease for a family member with dementia. Dementia 2009; 8: 95–116.

[66] Mukadam N , Cooper C , Basit B , Livingston G. Why do ethnic elders present later to UK dementia services? A qualitative study. Int Psychogeriatr 2011; 23: 1070–7. 21349212 10.1017/S1041610211000214

[67] Koehn S , McCleary L , Garcia L , Spence M , Jarvis P , Drummond N. Understanding Chinese–Canadian pathways to a diagnosis of dementia through a critical-constructionist lens. J Aging Stud 2012; 26: 44–54.

[68] McCleary L , Persaud M , Hum S , Pimlott NJ , Cohen CA , Koehn S , et al. Pathways to dementia diagnosis among South Asian Canadians. Dementia 2013; 12: 769–89. 24337639 10.1177/1471301212444806

[69] Garcia LJ , McCLeary L , Emerson V , Léopoldoff H , Dalziel W , Drummond N , et al. The pathway to diagnosis of dementia for francophones living in a minority situation. Gerontologist 2014; 54: 964–75. 24142913 10.1093/geront/gnt121

[70] Jackson S. Caregivers’ Perceptions of an Early Diagnosis of Alzheimer’s Disease in African Americans. Walden University, 2016 (https://scholarworks.waldenu.edu/dissertations/2290/).

[71] Czapka EA , Sagbakken M. ‘It is always me against the Norwegian system.’ barriers and facilitators in accessing and using dementia care by minority ethnic groups in Norway: a qualitative study. BMC Health Serv Res 2020; 20: 1–15. 10.1186/s12913-020-05801-6 PMC7565363 33059685

[72] Heng WAM , Lin YP , Chua WL , Chan E-Y. The early stages of caregiving: a qualitative study into the caregiving experiences of Asian family caregivers of persons with newly-diagnosed dementia. Geriatr Nurs 2021; 42: 1517–24. 34735998 10.1016/j.gerinurse.2021.10.015

[73] Blinka MD , Gundavarpu S , Baker D , Thorpe Jr RJ , Gallo JJ , Samus QM , et al. ‘At least we finally found out what it was’: dementia diagnosis in minoritized populations. J Am Geriatr Soc 2023; 71: 1952–62. 36914987 10.1111/jgs.18329 PMC10258149

[74] Grunberg VA , Bannon SM , Reichman M , Popok PJ , Vranceanu A-M. Psychosocial treatment preferences of persons living with young-onset dementia and their partners. Dementia 2022; 21: 41–60. 34151598 10.1177/14713012211027007 PMC10289008

[75] Van Vliet D , de Vugt ME , Bakker C , Koopmans RT , Pijnenburg YA , Vernooij-Dassen MJ , et al. Caregivers’ perspectives on the pre-diagnostic period in early onset dementia: a long and winding road. Int Psychogeriatr 2011; 23: 1393–404. 21729410 10.1017/S1041610211001013

[76] Hoppe S. Shifting uncertainties in the pre-diagnostic trajectory of early-onset dementia. Dementia 2019; 18: 613–29. 28081618 10.1177/1471301216687436

[77] Lai M , Jeon Y-H , McKenzie H , Withall A. Journey to diagnosis of young-onset dementia: a qualitative study of people with young-onset dementia and their family caregivers in Australia. Dementia 2023; 22: 1097–114. 37126513 10.1177/14713012231173013 PMC10262327

[78] Connell CM , Gallant MP. Spouse caregivers’ attitudes toward obtaining a diagnosis of a dementing illness. J Am Geriatr Soc 1996; 44: 1003–9. 8708288 10.1111/j.1532-5415.1996.tb01881.x

[79] Chrisp TAC , Tabberer S , Thomas BD. Bounded autonomy in deciding to seek medical help: carer role, the sick role and the case of dementia. J Health Psychol 2013; 18: 272–81. 22408064 10.1177/1359105312437265

[80] Aranda MP , Kremer IN , Hinton L , Zissimopoulos J , Whitmer RA , Hummel CH , et al. Impact of dementia: health disparities, population trends, care interventions, and economic costs. J Am Geriatr Soc 2021; 69: 1774–83. 34245588 10.1111/jgs.17345 PMC8608182

[81] Kenning C , Daker-White G , Blakemore A , Panagioti M , Waheed W. Barriers and facilitators in accessing dementia care by ethnic minority groups: a meta-synthesis of qualitative studies. BMC Psychiatry 2017; 17: 316. 28854922 10.1186/s12888-017-1474-0 PMC5577676

[82] Cooper C , Tandy AR , Balamurali TB , Livingston G. A systematic review and meta-analysis of ethnic differences in use of dementia treatment, care, and research. Am J Geriatr Psychiatry 2010; 18: 193–203. 20224516 10.1097/JGP.0b013e3181bf9caf

[83] Pham TM , Petersen I , Walters K , Raine R , Manthorpe J , Mukadam N , et al. Trends in dementia diagnosis rates in UK ethnic groups: analysis of UK primary care data. Clin Epidemiol 2018; 10: 949–60. 30123007 10.2147/CLEP.S152647 PMC6087031

[84] Nichols E , Steinmetz JD , Vollset SE , Fukutaki K , Chalek J , Abd-Allah F , et al. Estimation of the global prevalence of dementia in 2019 and forecasted prevalence in 2050: an analysis for the Global Burden of Disease Study 2019. Lancet Public Health 2022; 7: e105–25. 34998485 10.1016/S2468-2667(21)00249-8 PMC8810394

[85] Mattap SM , Mohan D , McGrattan AM , Allotey P , Stephan BC , Reidpath DD , et al. The economic burden of dementia in low-and middle-income countries (LMICs): a systematic review. BMJ Global Health 2022; 7: e007409. 10.1136/bmjgh-2021-007409 PMC8981345 35379735

[86] Sandberg LJ. Dementia and the gender trouble? Theorising dementia, gendered subjectivity and embodiment. J Aging Stud 2018; 45: 25–31. 29735206 10.1016/j.jaging.2018.01.004

[87] Morgan D , Innes A , Kosteniuk J. Dementia care in rural and remote settings: a systematic review of formal or paid care. Maturitas 2011; 68: 17–33. 21041045 10.1016/j.maturitas.2010.09.008

[88] Alzheimer’s Disease International. Dementia-Friendly Communities: Global Developments 2nd ed. Alzheimer’s Disease International, 2017.

[89] Moher D , Tsertsvadze A. Systematic reviews: when is an update an update? Lancet 2006; 367: 881–3. 16546523 10.1016/S0140-6736(06)68358-X

[90] Bowleg L. The problem with the phrase women and minorities: intersectionality – an important theoretical framework for public health. Am J Public Health 2012; 102: 1267–73. 22594719 10.2105/AJPH.2012.300750 PMC3477987

[91] Campbell F , Tricco AC , Munn Z , Pollock D , Saran A , Sutton A , et al. Mapping reviews, scoping reviews, and evidence and gap maps (EGMs): the same but different – the ‘Big Picture’ review family. Syst Rev 2023; 12: 45. 36918977 10.1186/s13643-023-02178-5 PMC10014395

[92] Waffenschmidt S , Knelangen M , Sieben W , Bühn S , Pieper D. Single screening versus conventional double screening for study selection in systematic reviews: a methodological systematic review. BMC Med Res Methodol 2019; 19: 1–9. 31253092 10.1186/s12874-019-0782-0 PMC6599339

[93] Bunn F , Sworn K , Brayne C , Iliffe S , Robinson L , Goodman C. Contextualizing the findings of a systematic review on patient and carer experiences of dementia diagnosis and treatment: a qualitative study. Health Expect 2015; 18: 740–53. 24286596 10.1111/hex.12162 PMC5060836

