# Supplementary material for: A mapping review of studies exploring the barriers and facilitators to a dementia diagnosis through an intersectionality lens
Source: BJPsych Open. 2025 Apr 11;11(3):e76. doi: 10.1192/bjo.2025.17 (PMC12052574; doi:10.1192/bjo.2025.17)
Supplement: Hicks et al. supplementary material [file S2056472425000171sup001.docx]

| **Supplementary Table 2.** Study characteristics of included studies | | | | | | | | |
| --- | --- | --- | --- | --- | --- | --- | --- | --- |
| **Author/Year/ Country** | **Aim of study** | **Sample** | **Sex** | **Average Age, years (Mean; SD)/(Median; IQR)** | **Predominant ethnicity, %age** | **Predominant dementia diagnosis, %age^1^** | **Design/Data collection** | **Key findings** |
| Chenoweth &  Spencer (1986)[^36^](#_ENREF_36)  US | To explore the experiences of families from the time of the earliest recognition of symptoms of dementia throughout the course of the illness | 289 family members | 76.8% female | M = NR (SD= NR) | 99% White | 91.7% AD | Mixed methods – mail survey with  open and closed questions and telephone interviews | Barriers:  Preserving autonomy, Problems accessing help  Facilitators:  Recognition of symptoms as a problem |
| Connell & Gallant (1996)[^78^](#_ENREF_78)  US | To examine attitudes of spouse caregivers about the process of obtaining a diagnosis of a dementing illness | 233 spousal carers | 53% female | M = 68 (SD= NR) | 91% White | 54% AD | Mixed methods – mail survey with  closed and open questions | Barriers:  Normalisation of symptoms, Lack of perceived need, Denial, Preserving autonomy, Lack of informal network support, Problems accessing help |
| Boise et al. (1999)[^37^](#_ENREF_37)  US | To determine time periods between the initial recognition by family members of symptoms of memory loss and seeking medical consultation and factors attributed to a delay in diagnosis | 53 carers | 67% female | M= 54 (SD= NR) | NR | NR | Mixed methods – focus groups and mail survey | Barriers:  Normalisation of symptoms, Denial, Preserving autonomy, Lack of informal network support, Lack of knowledge, Problems accessing help, Stigma and fear, Unaware of changes, Carer difficulties  Facilitators:  Recognition of symptoms as a problem |
|  |  | 191 carers | NR | NR (NR) | NR | 67% AD |  |  |
| Knopman et al. (2000)[^38^](#_ENREF_38)  US | Description of factors associated with delays in diagnosis of Alzheimer's disease | 1480 carers | 52.4% female | M = 58 (SD= NR) | 88% White | 100% AD | Quantitative - mail survey | Barriers:  Normalisation of symptoms, Lack of perceived need, Denial, Preserving autonomy, Lack of informal network support  Facilitators:  Recognition of symptoms as a problem |
| Ortiz & Fitten (2000)[^60^](#_ENREF_60)  US | To explore perceived barriers to healthcare access in cognitively impaired older Hispanic patients | 65 carers | 58% female | M = 68 (SD = 9) | 100% Hispanic | NR | Quantitative – structured interview using the Barriers to Healthcare Access Survey | Barriers:  Normalisation of symptoms, Preserving autonomy, Problems accessing help, Stigma and fear |
| Wackerbarth & Johnson (2002)[^39^](#_ENREF_39)  Streams, Wackerbarth  & Maxwell (2003)[^40^](#_ENREF_40)  US | To identify the benefits and barriers perceived by family caregivers of persons who have been through a diagnostic assessment for dementia symptoms | 528 carers** | 67.8% female | M=61.3 (SD = NR) | NR | 67.8% AD | Mixed methods – Survey with  closed and open questions | Barriers:  Normalisation of symptoms, Lack of perceived need, Denial, Preserving autonomy, Lack of information network support, Lack of knowledge, Problems accessing help, Stigma and fear, Unaware of changes, Carer difficulties  Facilitators:  Recognition of symptoms as a problem, Prior knowledge and contacts |
| Cloutterbuck & Mahoney (2003)[^61^](#_ENREF_61)  US | To explore the perceptions and  experiences of African American caregivers on their journey toward a diagnosis of dementia for their loved ones | 7 carers | 71.4% female | NR (NR) | 100% African American | NR | Qualitative – focus group | Barriers:  Normalisation of symptoms, Preserving autonomy, Lack of knowledge  Facilitators:  Recognition of symptoms as a problem, Support from informal network, Prior knowledge and contacts |
| Teel and Carson (2003)[^41^](#_ENREF_41)  US | To describe the experience of families in seeking diagnosis and treatment for loved one with dementia | 14 carers | 64% female | M = 62.4 (SD= 12.1)* | 100% White | NR | Qualitative – semi structured interviews | Barriers:  Lack of knowledge  Facilitators:  Recognition of symptoms as a problem |
| Zhan (2004)[^62^](#_ENREF_62)  US | To examine the experiences of Chinese American Caregivers who provide care for family members with AD and factors that  hinder or facilitate obtaining an AD diagnosis | 4 carers | 75% female | NR | 100% Chinese American | 100% AD | Qualitative - Semi structured interviews | Barriers:  Lack of knowledge, Problems accessing help, Stigma and fear  Facilitators: Prior knowledge and contacts |
| Clark et al. (2005)[^63^](#_ENREF_63)  US | To identify early patterns of care for AD in a cohort of African American patients and their caregivers presenting at an inner  city clinic and a suburban memory  assessment clinic | 79 carers | 84.8% female | M = 75.3 (SD = 8.2)* | 100% African American | 100% AD | Quantitative – structured interview | Barriers:  Normalisation of symptoms, Lack of perceived need, Denial, Preserving autonomy  Facilitators:  Recognition of symptoms as a problem |
| Krull (2005)[^42^](#_ENREF_42)  US | To explore the process through which familial caregivers decide to seek out a formal diagnosis of Alzheimer’s disease in their loved ones | 13 carers | 61.5% female | NR | 100% White | NR | Qualitative, interactionist framework – semi-structured interviews | Barriers:  Normalisation of symptoms  Facilitators:  Recognition of symptoms as a problem, Prior knowledge and contacts |
| Neary & Mahoney (2005)[^64^](#_ENREF_64)  US | To explore the experience of dementia caregiving in an ethnically diverse sample of Latino caregivers | 11 carers | 81.8% female | M = 50 (NR) | 100% Hispanic | NR | Qualitative - Semi structured interviews | Barriers:  Normalisation of symptoms, Denial, Lack of knowledge  Facilitators:  Recognition of symptoms as a problem, Prior knowledge and contacts |
| Rimmer et al. (2005)[^43^](#_ENREF_43)  Bond et al. (2005)[^44^](#_ENREF_44)  Europe | To assess attitudes and behaviours towards Alzheimer’s Disease in Europe | 618 carers | NR | NR | NR | 100% AD | Mixed methods – Carers Quantitative interview survey, PLWD qualitative interviews | Barriers:  Normalisation of symptoms, Lack of perceived need, Denial, Lack of knowledge  Facilitators:  Recognition of symptoms as a problem |
|  |  | 96 PLWD | NR | M = 74 (SD=NR) | NR | 100% AD |  |  |
| Eustace et al. (2007)[^45^](#_ENREF_45)  Ireland | To determine the frequency of unrecognised dementia in a group of community dwelling elderly and identify factors associated with dementia recognition | 62 carers | NR | NR | NR | 64.5% AD | Quantitative – structured interview | Barriers:  Normalisation of symptoms, Lack of perceived need, Preserving autonomy, Problems accessing help  Facilitators:  Recognition of symptoms as a problem |
| Carpentier et al. (2008)[^46^](#_ENREF_46)  Canada | To analyse service use barriers using the social representation approach | 52 carers | 78.8% female | M= 66.0 (SD=NR) | NR | 100% AD | Qualitative, Social representation  approach - Semi structured interviews | Barriers:  Normalisation of symptoms, Lack of informal network support, Stigma and fear  Facilitators:  Recognition of symptoms as a problem, Support from informal network, Prior knowledge and contacts |
| Speechly et al. (2008)[^47^](#_ENREF_47)  Australia | To describe the steps taken and delays encountered in the pathway to dementia diagnosis | 209 carers | 75% female | M = 65.5 (SD=12.2) | NR | 58% AD | Quantitative – mail survey | Barriers:  Normalisation of symptoms, Lack of perceived need, Denial, Preserving autonomy |
| Hughes et al. (2009)[^65^](#_ENREF_65)  US | To examine the experiences of African American caregivers seeking a formal diagnosis for a family member with chronic memory loss | 17 carers | 82.4% female | M= 59.8 | 100% African American | NR | Qualitative using the Health Belief  Model – semi structured interviews | Barriers:  Normalisation of symptoms, Denial, Lack of informal network support  Facilitators:  Recognition of symptoms as a problem, Support from informal network |
| Tsolaki et al. (2009)[^48^](#_ENREF_48)  Greece | To assess the attitudes and perceptions of physicians, caregivers and the general public towards Alzheimer’s Disease in Greece | 100 carers | NR | NR | NR | NR | Quantitative – telephone survey | Barriers:  Lack of perceived need, Denial, Lack of knowledge  Facilitators:  Recognition of symptoms as a problem |
| Carpentier et  al. (2010)[^49^](#_ENREF_49)  Canada | To analyse the initial period of Alzheimer’s disease (the period that starts with the first manifestations of the illness and ends with the diagnosis). | 60 carers | 71.7% female | NR | NR | NR | Qualitative, Life course  perspective - interviews | Barriers:  Normalisation of symptoms, Lack of perceived need, Denial, Lack informal network support, Unaware of changes, Carer difficulties  Facilitators:  Recognition of symptoms as a problem, Support from informal network |
| Jones et al. (2010)[^50^](#_ENREF_50)  Europe | To update current knowledge on the attitudes and behaviours towards Alzheimer’s Disease in Europe | 250 carers | NR | NR | NR | 100% AD | Quantitative – online questionnaire | Barriers:  Normalisation of symptoms, Lack of perceived need, Denial, Preserving autonomy, Lack informal network support  Facilitators:  Recognition of symptoms as a problem |
| Leung et al. (2011)[^51^](#_ENREF_51)  Canada | To retrospectively explore the experiences of Anglo-Canadians with dementia and their carers regarding the sequence of symptoms and events that preceded the diagnosis | 6 PLWD | 33.3% female | NR | 100% Angalo-canadian | 66.6% AD | Qualitative/ Phenomenology  - Semi-structured interviews | Barriers:  Normalisation of symptoms, Denial  Facilitators:  Recognition of symptoms as a problem, Support from informal network |
|  |  | 7 carers | 71.4% female | NR | 100% Angalo-canadian | NR |  |  |
| Manthorpe et al. (2011)[^52^](#_ENREF_52)  UK | To understand the experiences, expectations and service needs of the person who is becoming the person with dementia from the perspectives of the older person and their supporter or carer | 27 PLWD | 51.9% female | NR (NR) | 96.3% White British | NR | Qualitative – interview with topic guide | Barriers:  Normalisation of symptoms, Lack of perceived need, Denial, Lack informal network support  Facilitators:  Recognition of symptoms as a problem, Support from informal network, Prior knowledge and contacts |
|  |  | 26 carers | 76.9% Female | NR (NR) | 96.3% White British | NR |  |  |
| Mukadam et al. (2011)[^66^](#_ENREF_66)  UK | Explore the effect of culture and ethnicity on beliefs and attitudes of carers to help-seeking for dementia symptoms | 18 carers | 72.2% female | M = 57 (NR) | 27.8% South Asian and 27.8% Black African | NR | Qualitative – semi-structured interviews | Barriers:  Normalisation of symptoms, Lack of perceived need, Denial, Preserving autonomy, Lack informal network support, Problems accessing help, Stigma and fear  Facilitators:  Recognition of symptoms as a problem |
| Van Vliet et al. (2011)[^75^](#_ENREF_75)  Netherlands | To investigate the barriers to obtaining a diagnosis for caregivers of people with early onset dementia | 92 carers | 52.2% female | M=59.3 (8.7) | NR | 68% AD | Qualitative, grounded theory – semi structured interviews | Barriers:  Normalisation of symptoms, Lack of perceived need, Denial, Preserving autonomy, Lack informal network support, Unaware of changes  Facilitators:  Recognition of symptoms as a problem, Support from informal network |
| Chrisp et al.  (2012)[^53^](#_ENREF_53)  Chrisp et al.  (2012)[^79^](#_ENREF_79)  UK | To identify factors at different points in the journey that delay and facilitate first contact with an HCP. | 20 carers | 65% female | NR | 100% White British | NR | Qualitative - interviews | Barriers:  Normalisation of symptoms, Lack of perceived need, Denial, Preserving autonomy, Lack informal network support, Carer difficulties  Facilitators:  Recognition of symptoms as a problem, Support from informal network |
| Koehn et al. (2012)[^67^](#_ENREF_67)  Canada | To study the pathway to a diagnosis of dementia as experienced by ten dyads of people with Chinese origin | 10 PLWD | 20% female | NR (NR) | 100% Chinese-Canadian | NR | Qualitative, critical constructionist and intersectionality lens – semi structured interviews | Barriers:  Normalisation of symptoms  Facilitators:  Recognition of symptoms as a problem, Support from informal network, Prior knowledge and contacts |
|  |  | 11 carers | 81.9% female | NR (NR) | 100% Chinese-Canadian | NR |  |  |
| McCleary et al. (2012)[^68^](#_ENREF_68)  Canada | To explore experiences of South Asian Canadian persons with dementia and their family carers in the time prior to a diagnosis of dementia | 6 PLWD | 66.7% female | M= 80 (SD= NR) | 100% South Asian | NR | Qualitative – semi-structured interviews | Barriers:  Normalisation of symptoms, Lack of perceived need, Lack of knowledge  Facilitators:  Recognition of symptoms as a problem, Support from informal network, Prior knowledge and contacts |
|  |  | 8 carers | 63% female | NR | 100% Canadian born South Asians | NR |  |  |
| Bunn et al. (2013)[^93^](#_ENREF_93)  UK | To test and contextualise the findings of a systematic review of qualitative studies looking at patient and carer experiences of diagnosis and treatment of dementia | 3 PLWD | 33.3% female | NR | NR | NR | Qualitative - focus groups and one to one interviews | Barriers:  Normalisation of symptoms, Lack of knowledge, Stigma and fear  Facilitators:  Recognition of symptoms as a problem |
|  |  | 12 carers | 58.3% female | NR | NR | NR |  |  |
| Garcia et al. (2013)[^69^](#_ENREF_69)  Canada | To describe the experiences associated with the pathway to dementia diagnosis among francophone Canadians | 7 PLWD | 57.1% female | M= 73.4 (SD=7.2) * | NR | NR | Qualitative - interviews | Barriers:  Normalisation of symptoms, Lack of perceived need, Preserving autonomy, Lack of informal network support, Lack of knowledge  Facilitators:  Recognition of symptoms as a problem, Support from informal network |
|  |  | 7 Carers | 71.4% female | NR | NR | NR |  |  |
| Jackson (2016)[^70^](#_ENREF_70)  US | To identify the barriers to accurate and early diagnosis of Alzheimer’s Disease among African Americans from the  perspective of family caregivers | 8 carers | 75% female | M= 59.6 (13.4) | 100% African American | 100% AD | Qualitative, grounded theory  using the health belief model and attribution theory as a theoretical  framework – Semi-structured interviews | Barriers:  Normalisation of symptoms, Lack of perceived need, Denial, Preserving autonomy, Lack of informal network support, Unaware of changes, Carer difficulties  Facilitators:  Recognition of symptoms as a problem, Support from informal network |
| Feldman et al. (2017)[^55^](#_ENREF_55)  UK | To examine family carers accounts of first onset and symptom attribution when they first became concerned about their relative’s early signs of dementia | 84 carers | 75% female | M = 66 (SD= 12.6) | 87% White UK | AD 35% | Mixed methods - Semi-structured  interviews with closed and open questions | Barriers:  Normalisation of symptoms, Lack of perceived need  Facilitators:  Recognition of symptoms as a problem, Prior knowledge and contacts |
| Lian et al. (2017)[^34^](#_ENREF_34)  China | To understand the experiences of people with dementia and their caregivers in engaging in dementia diagnosis | 20 carers | 80% female | M= 58.0 (SD = 15.1) | NR | NR | Qualitative/ Gadamer’s Hermeneutic principles and social ecological theory – Focus group and one to one interviews | Barriers:  Normalisation of symptoms, Lack of perceived need, Problems accessing help, Stigma and fear  Facilitators:  Recognition of symptoms as a problem, Prior knowledge and contacts |
|  |  | 3 PLWD | NR | NR | NR | NR |  |  |
| Woods et al. (2018)[^11^](#_ENREF_11)  Europe | To examine to what extent timely diagnoses of dementia are occurring across Europe, what factors are associated with it, and what is the impact on carers emotions of quality of diagnostic disclosure | 1,409 family carers | 83% female | Mdn = 57 (IQR= NR) | NR | NR | Quantitative survey | Barriers:  Normalisation of symptoms, Denial, Problems accessing help |
| Hoppe (2019)[^76^](#_ENREF_76)  Netherlands | To examine how the pre-diagnostic process is perceived by people with early-onset dementia and their family members | 7 PLWD | 57% female | NR | NR | NR | Qualitative - interviews | Barriers:  Normalisation of symptoms, Denial, Unaware of changes  Facilitators:  Recognition of symptoms as a problem, Support from informal network, Prior knowledge and contacts |
|  |  | 39 family caregivers | 77% female | NR | NR | NR |  |  |
| Czapka & Sagbakken (2020)[^71^](#_ENREF_71)  Norway | To explore the barriers and facilitators in accessing and using dementia care services by minority ethnic groups in Norway | 11 family carers | NR | NR | 100% “minority ethnic groups” | NR | Qualitative - Semi-structured in-depth interviews | Barriers:  Normalisation of symptoms, Lack of perceived need, Lack of knowledge, Problems accessing help, Stigma and fear  Facilitators:  Recognition of symptoms as a problem, Support from informal network |
| Willis et al., (2020)[^35^](#_ENREF_35)  Pakistan | To explore the experiences of dementia in Pakistan | 20 PLWD with carer support | 45% female | NR | 100% Pakistani | NR | Qualitative - interviews | Facilitators:  Recognition of symptoms as a problem, Support from informal network |
| Heng et al (2021)[^72^](#_ENREF_72)  Singapore | Examine early experiences of Asian family caregivers focussing on diagnosis journey and immediate adjustments post-diagnosis | 11 family carers | 73% female | M= 62.5 years (SD=14.5) | 82% Chinese and | AD 91% | Qualitative- semi-structured interviews | Barriers:  Normalisation of symptoms, Lack of knowledge  Facilitators:  Recognition of symptoms as a problem, Support from informal network, Prior knowledge and contacts |
| Novek & Menec (2021)[^21^](#_ENREF_21)  Canada | To examine the process of accessing and delivering a diagnosis of young onset dementia | 6 PLWD | 33.3% female | NR (NR) | 100% White Canadian | AD 66.7% | Semi-structured interviews using the candidacy framework | Barriers:  Normalisation of symptoms, Denial, Lack of knowledge  Facilitators:  Recognition of symptoms as a problem |
|  |  | 14 family carers | 50% female | NR (NR) | 100% White Canadian | AD 40% |  |  |
| Grunberg et al. (2022)[^74^](#_ENREF_74)  US | To gain an in-depth understanding of the psychosocial treatment preferences of persons with young-onset dementia and their partners | 23 PLWD | 48% female | M= 61.3 (SD= 4.7) | 87% White non-Hispanic or Latino | Atypical AD 35% | Dyadic qualitative interviews | Barriers:  Normalisation of symptoms, Denial, Preserving autonomy, Lack of knowledge, Unaware of changes  Facilitators:  Recognition of symptoms as a problem, Support from informal network, Prior knowledge and contacts |
|  |  | 23 spouses | 57% female | M= 60.5 years (SD=5.4) | 91% White non-Hispanic or Latino | Atypical AD 35% |  |  |
| Brady et al (2022)[^56^](#_ENREF_56)  Ireland | Explore views on the impact of the timing of dementia diagnosis to identify barriers to early diagnosis | 1 PLWD | NR | NR | NR | NR | Qualitative- semi-structured interviews with content analysis | Barriers:  Normalisation of symptoms, Stigma and fear  Facilitators:  Recognition of symptoms as a problem |
|  |  | 7 family members | NR | NR | NR | NR |  |  |
| Parker et al (2022)[^57^](#_ENREF_57)  UK | Explore experiences of carers of people diagnosed with dementia during or following a hospital admission, to identify factors that prevented help-seeking beforehand | 12 carers | 92% female | NR | NR | NR | Qualitative- semi-structured interviews | Barriers:  Normalisation of symptoms, Lack of perceived need, Denial, Unaware of changes, Lack of knowledge, Lack of informal network support, Problems accessing help, Stigma and fear, Preserving autonomy, Carer difficulties  Facilitators:  Recognition of symptoms as a problem, Prior knowledge and contacts |
| Blinka et al (2023)[^73^](#_ENREF_73)  US | Identify factors delaying or facilitating diagnosis in minoritised people with dementia and elicit care partner perspectives on the timing and effects of diagnosis | 19 carers | 83% female | M= 61.6 years (SD=9.25)* | 61% Black/ African American | NR | Qualitative- semi-structured interviews | Barriers: Lack of knowledge, Normalisation of symptoms, Lack of informal network support, Stigma and fear, Problems accessing help  Facilitators:  Recognising symptoms as a problem |
| Lai et al., (2023)[^77^](#_ENREF_77)  Australia | Explore the journey to diagnosis and subsequent reactions to the diagnosis of young-onset dementia from people living with the condition and family carers | 28 family carers | 60.7% female | M= 52.3 years (SD= 13.5) | NR | NR | Qualitative- semi-structured interviews | Barriers:  Denial, Normalisation of symptoms, Lack of knowledge, Stigma and fear  Facilitators:  Recognising symptoms as a problem |
|  |  | 14 PLWD | 64% male | M= 62.2 years (SD= 3) | NR | AD 57.1% |  |  |
| Sideman et al (2023)[^58^](#_ENREF_58)  US | Examine the experiences of the diagnostic process for sporadic Creutzfeldt-Jakob disease from perspective of family carers | 12 family carers | 50% female | M= 59 years (SD= 7)* | 75% White | sporadic Creutzfeldt-Jakob 100% | Qualitative- in-depth interviews | Barriers:  Lack of knowledge  Facilitators:  Recognising symptoms as a problem |
| Acton et al (2024)[^59^](#_ENREF_59)  UK | Examine the challenges in caring for a person with intellectual disability and the barriers to obtaining a diagnosis of dementia | 14 formal/paid carers | 71.4% female | NR | NR | NR | Qualitative- in-depth interviews with IPA | Barriers:  Normalisation of symptoms, Lack of knowledge |
| 1. When the sample is composed of carers, the value reflects the predominant diagnosis of care recipients.   *Values calculated based on available data. **Study characteristics derived from the complete sample from Wackerbarth & Johnson, 2002 (n=528), rather than the subset of participants reported in Streams et al., 2013 (n= 416).  AD = Alzheimer’s disease , NR = Not reported, M = Mean, PLWD = People living with dementia, SD= Standard deviation, Mdn = Median, IQR = Interquartile Range. | | | | | | | | |

| Supplementary Table A. Example search strategies for two databases that will be used in mapping review. | |
| --- | --- |
| Database | PsychINFO |
| Terms used | ti((dement* OR Alzheimer* OR ("lewy bodies" OR "lewy body") OR "memory problem" OR ("cognitive disorder" OR "cognitive disorders") OR "neurocognitive disorder*" OR confus* OR forgetful*)) AND ab(diagnosis OR "early diagnosis" OR delay* diagnosis OR "late diagnosis" OR undiagnosed OR undetected) AND ab(("help seeking" OR "help-seeking" OR seek*OR access* OR delay* OR avoid* OR barrier* OR facilitat*  OR enable* OR trigger* OR obstacle*)) NOT (Peptide OR in-vivo OR mouse OR mice OR MRI OR PET OR biomarkers OR animal OR gene OR genotype) |

Supplementary Table B. The number of records in which barriers and facilitators are reported through a specific social lens. Percentages are reported out of a total of 56 records.

|  | Barriers | | | | | | | | | | | Facilitators | | | |
| --- | --- | --- | --- | --- | --- | --- | --- | --- | --- | --- | --- | --- | --- | --- | --- |
|  | Normalisation of symptoms | Lack of perceived need | Denial | Preserving autonomy | Lack of informal network support | Lack of knowledge | Problems accessing help | Stigma and fear | Unaware of changes | Carer difficulties | Recognition of symptoms as a problem | | Support from informal network | Prior knowledge and contacts |  |
| Familial | 2 (3.6%) | 1 (1.8%) | 2 (3.6%) | 1 (1.8%) | 1 (1.8%) | 0 (0.0%) | 2 (3.6%) | 0 (0.0%) | 0 (0.0%) | 0 (0.0%) | 0 (0.0%) | | 0 (0.0%) | 0 (0.0%) |  |
| Age | 6 (10.7%) | 1 (1.8%) | 5 (8.9%) | 2 (3.6%) | 1 (1.8%) | 3 (5.4%) | 0 (0.0%) | 0 (0.0%) | 3 (5.4%) | 0 (0.0%) | 6 (10.7%) | | 4 (7.1%) | 2 (3.6%) |  |
| Ethnicity | 13 (23.2%) | 6 (10.7%) | 5 (8.9%) | 6 (10.7%) | 5 (8.9%) | 8 (14.3%) | 5 (8.9%) | 5 (8.9%) | 1 (1.8%) | 1 (1.8%) | 3 (5.4%) | | 9 (16.1%) | 7 (12.5%) |  |
| Nationality | 1 (1.8%) | 0 (0.0%) | 1 (1.8%) | 0 (0.0%) | 0 (0.0%) | 0 (0.0%) | 1 (1.8%) | 0 (0.0%) | 0 (0.0%) | 0 (0.0%) | 0 (0.0%) | | 0 (0.0%) | 0 (0.0%) |  |
| Non-specific | 20 (35.7%) | 14 (25.0%) | 13 (23.2%) | 9 (16.1%) | 8 (14.3%) | 10 (17.9%) | 6 (10.7%) | 7 (12.5%) | 4 (7.1%) | 5 (8.9%) | 22 (39.3%) | | 5 (8.9%) | 7 (12.5%) |  |
| Gender | 2 (3.6%) | 1 (1.8%) | 1 (1.8%) | 1 (1.8%) | 1 (1.8%) | 0 (0.0%) | 0 (0.0%) | 0 (0.0%) | 1 (1.8%) | 1 (1.8%) | 2 (3.6%) | | 2 (3.6%) | 1 (1.8%) |  |
| Socio-economic status | 1 (1.8%) | 1 (1.8%) | 0 (0.0%) | 0 (0.0%) | 0 (0.0%) | 1 (1.8%) | 1 (1.8%) | 1 (1.8%) | 0 (0.0%) | 0 (0.0%) | 1 (1.8%) | | 2 (3.6%) | 2 (3.6%) |  |
| Culture | 3 (5.4%) | 2 (3.6%) | 1 (1.8%) | 1 (1.8%) | 1 (1.8%) | 1 (1.8%) | 2 (3.6%) | 2 (3.6%) | 0 (0.0%) | 0 (0.0%) | 2 (3.6%) | | 2 (3.6%) | 2 (3.6%) |  |
| Rurality | 0 (0.0%) | 0 (0.0%) | 0 (0.0%) | 0 (0.0%) | 0 (0.0%) | 0 (0.0%) | 0 (0.0%) | 0 (0.0%) | 0 (0.0%) | 0 (0.0%) | 0 (0.0%) | | 0 (0.0%) | 0 (0.0%) |  |
